# Photosynthetic Cost Associated With Induced Defense to *Plasmopara viticola* in Grapevine

**DOI:** 10.3389/fpls.2020.00235

**Published:** 2020-03-19

**Authors:** Antonio F. Nogueira Júnior, Merle Tränkner, Rafael V. Ribeiro, Andreas von Tiedemann, Lilian Amorim

**Affiliations:** ^1^Department of Plant Pathology, ESALQ, University of São Paulo, Piracicaba, Brazil; ^2^Department of Crop Sciences, Institute of Applied Plant Nutrition, University of Göttingen, Göttingen, Germany; ^3^Department of Plant Biology, Institute of Biology, University of Campinas, Campinas, Brazil; ^4^Department of Crop Sciences, Division of Plant Pathology and Crop Protection, University of Göttingen, Göttingen, Germany

**Keywords:** gas exchange, downy mildew, *Vitis vinifera*, photosynthesis limitations, biotic stress

## Abstract

Downy mildew caused by *Plasmopara viticola* is one of the most destructive diseases of *Vitis vinifera* worldwide. Grapevine breeding programs have introgressed *P. viticola*-resistant traits into cultivated *V. vinifera* genotypes and launched interspecific hybrids with resistance against downy mildew. In general, pathogen infection affects primary metabolism, reduces plant growth and development and modifies the secondary metabolism toward defense responses, which are costly in terms of carbon production and utilization. The objective of this work was to evaluate the photosynthesis impairment by inducible defenses at the leaf level in *V. vinifera* cultivars resistant to *P. viticola*. Photosynthetic limitations imposed by *P. viticola* in susceptible and resistant grapevine cultivars were evaluated. Histochemical localization of hydrogen peroxide and superoxide and the activity of ascorbate peroxidase were assessed. Measurements of leaf gas exchange, chlorophyll fluorescence and the response of leaf CO_2_ assimilation to increasing air CO_2_ concentrations were taken, and photosynthetic limitations determined in cultivars Solaris (resistant) and Riesling (susceptible). The net photosynthetic rates were reduced (−25%) in inoculated Solaris plants even before the appearance of cell death-like hypersensitive reactions (“HR”). One day after “HR” visualization, the net photosynthetic rate of Solaris was reduced by 57% compared with healthy plants. A similar pattern was noticed in resistant Cabernet Blanc and Phoenix plants. While the susceptible cultivars did not show any variation in leaf gas exchange before the appearance of visual symptoms, drastic reductions in net photosynthetic rate and stomatal conductance were found in diseased plants 12 days after inoculation. Decreases in the maximum Rubisco carboxylation rate and photochemical impairment were noticed in Riesling after inoculation with *P. viticola*, which were not found in Solaris. Damage to the photochemical reactions of photosynthesis was likely associated with the oxidative burst found in resistant cultivars within the first 24 h after inoculation. Both chlorophyll degradation and stomatal closure were also noticed in the incompatible interaction. Taken together, our data clearly revealed that the defense response against *P. viticola* causes a photosynthetic cost to grapevines, which is not reversible even 12 days after the pathogen infection.

## Introduction

Downy mildew, caused by the biotrophic oomycete *Plasmopara viticola*, is one of the most devastating diseases of grapevine. *P. viticola* is native to North America and was considered to be of minor importance on *Vitis aestivalis*, in which it was first described in 1834 (Wilcox et al., 2015). However, this pathogen was inadvertently introduced into Europe in 1878 and now it is endemic in the main grapevine growing regions worldwide (Gessler et al., 2011). The European grapevine (*Vitis vinifera*) evolved in the absence of this disease and it is extremely susceptible to *P. viticola*. After *P. viticola* introduction the pathogen quickly spread throughout the European vineyards (Rossi et al., 2013). *P. viticola* affects grapevine leaves, inflorescences, berries and shoots with high disease severity causing defoliation and producing low-quality, unsightly or entirely damaged grapes. The pathogen can also cause weakening, dwarfing and death of young shoots (Agrios, 2005). When the weather is conducive to the disease and in the absence of effective disease control, downy mildew can easily reduce the grapevine yield up to 100% (Agrios, 2005; Wilcox et al., 2015). *P. viticola* zoospores penetrate the host through stomata and develop intercellular mycelium in the mesophyll of grapevine leaves, generating globose haustoria responsible for the oomycete nutrition. After infection and colonization periods, sporangiophores and sporangia emerge from the stomata (Ash, 2000; Agrios, 2005; Gessler et al., 2011; Kamoun et al., 2015; Armijo et al., 2016). Currently, downy mildew is of paramount importance in all humid parts of the world where grapevine is grown. In subtropical regions of South America, the occurrence of rainy periods at the time of budbreak and flowering can lead to severe epidemics of downy mildew. Consequently, high frequency of fungicide sprays is necessary to control downy mildew during the rainy season, i.e., from summer to autumn (Cappello et al., 2017).

Although European *V. vinifera* cultivars are highly susceptible to downy mildew, *Muscadinia* species and several American and Asian *Vitis* species exhibit varying levels of resistance to *P. viticola*. By the conventional breeding, *V. vinifera* has been crossed with grapevines showing resistance against *P. viticola*, and resistant interspecific hybrids have been subsequently found. In resistant accessions obtained by crosses with North American *Vitis* species, *P. viticola* completes its life cycle and releases less sporangia than on susceptible cultivars (Bellin et al., 2009). The genomic regions conferring resistance to downy mildew referred to as resistance to *P. viticola* (*Rpv*) are quantitatively inherited. To date, 27 quantitative trait loci (QTLs) with major effects on downy mildew resistance are known and described (Fischer et al., 2004; Welter et al., 2007; Bellin et al., 2009; Marguerit et al., 2009; Vezzulli et al., 2019). *Rpv1* and *Rpv2* are responsible for the resistance derived from *Muscadinia rotundifolia* (Peressotti et al., 2010) and map to chromosomes 12 and 18, respectively (Merdinoglu et al., 2003; Welter et al., 2007). The locus *Rpv3*, which was first described in cultivars Regent and Bianca, came from the American wild *Vitis* species and is located on chromosome 18 (Fischer et al., 2004; Welter et al., 2007; Bellin et al., 2009). The Asian resistance loci *Rpv8*, *Rpv10*, and *Rpv12* originated from *Vitis amurensis*. *Rpv8* and *Rpv12* map to chromosome 14 (Venuti et al., 2013) and *Rpv10* maps to chromosome 9 and is present in cultivar Solaris (Blasi et al., 2011; Schwander et al., 2012; Venuti et al., 2013). The table of loci traits in grapevine relevant for breeding and genetics with a complete description of associated markers, their chromosomal localization, and the donor genotype/species of *P. viticola* resistance is available online (www.vivc.de/loci).

The mechanisms by which grapevine cells reduce downy mildew intensity are complex and not fully elucidated (Kortekamp, 2006; Liu et al., 2015). These mechanisms comprise pre-existing chemical defenses and inducible structural and biochemical defenses. High constitutive levels of antimicrobial compounds, such as inositol and caffeic acid, are observed in healthy leaves of resistant cultivars (Figueiredo et al., 2008). The induced structural defenses include formation of callose (Kortekamp et al., 1997; Gindro et al., 2003) and lignification (Dai et al., 1995). The accumulation of stilbenes and pathogenesis-related proteins (Kortekamp, 2006; Richter et al., 2006; Slaughter et al., 2008), the generation of reactive oxygen species, the induction of peroxidases (Kortekamp and Zyprian, 2003), and finally, cell death-like hypersensitive reactions (“HR”) represent induced biochemical defenses found in grapevine resistant cultivars after inoculation with *P. viticola* (Díez-Navajas et al., 2008).

Pathogen infection in incompatible interactions may affect the plant primary and also the secondary metabolism due to the induction of defenses (Berger et al., 2007). In the incompatible interaction of *Phytophthora nicotianae* and *Nicotiana tabacum*, photosynthesis is switched off few hours after pathogen inoculation, and defense mechanisms are initiated. This metabolic shift is related to an early blockage of intercellular sugar transport by callose deposition and increases in apoplastic invertase activity (Scharte et al., 2005). Both constitutive and inducible defenses are costly in terms of carbohydrate production and utilization. However, inducible systems seem to be less costly than constitutive systems in the absence of pathogens because inducible defenses require allocation of both energy and resources away from growth and reproduction (Cipollini, 1998; DeWitt et al., 1998; Purrington, 2000; Heil and Baldwin, 2002). The cost of inducible defenses is a widely discussed topic among breeders and pathologists and there are few papers conveying a significant piece of novel information on the drawbacks of plant disease resistance. The development of new resistant varieties to *P. viticola* is a promising way to control downy mildew and the better understanding of the effect of *P. viticola* in the leaf carbon assimilation of resistant plants is an important issue for the scientific community interested in grapevine-pathogen interaction.

*Plasmopara viticola* affects the photosynthesis of susceptible grapevine cultivars by reducing the diffusion of CO_2_ in leaf mesophyll (Jermini et al., 2010; Nogueira Júnior et al., 2019). In diseased plants, the concentration of photosynthetic pigments in areas nearby lesions and the primary photochemistry are reduced (Moriondo et al., 2005). Reductions and abundance in Rubisco activity have also been reported in susceptible cultivars of *V. vinifera* and *Vitis labrusca* infected by *P. viticola* (Gamm et al., 2011; Nogueira Júnior et al., 2019). However, the effect of downy mildew on photosynthetic capacity and leaf gas-exchange for resistant cultivars has not been characterized. Here, alterations in gas exchange, chlorophyll fluorescence and production of oxygen reactive species of leaves from cultivars with different resistance levels to downy mildew were examined. The objective of this work was to estimate the impairment of photosynthesis caused by inducible responses to the infection of *P. viticola* on downy mildew resistant cultivars and to elucidate the underlying limitations imposed by *P. viticola* to the photosynthesis of resistant and susceptible cultivars. The oxidative burst induced by *P. viticola* was also evaluated and related to the photosynthetic limitations in resistant cultivars.

## Materials and Methods

The characterization of photosynthetic cost associated with inducible defenses to *P. viticola* in grapevine was performed in three steps. The first step, which is described in section “Gas Exchange Variables in Cultivars Resistant and Susceptible to Downy Mildew,” aimed to evaluate gas exchange in grapevine leaves of different cultivars, resistant and susceptible to downy mildew. The resistant cultivars Solaris, Cabernet Blanc and Phoenix and the susceptible cultivars Riesling, Niagara Rosada, Merlot and Moscato were used in this step. For the second step two representative cultivars, one resistant (Solaris) and one susceptible (Riesling) to *P. viticola* were selected. This step aimed to evaluate the diffusive, photochemical and biochemical limitations imposed by *P. viticola* on grapevine photosynthesis. The experiment at the last step aimed to characterize some induced responses in the cultivar Solaris and to correlate these responses with the early decrease on photosynthesis of leaves challenged with *P. viticola*.

### Plant Material and Inoculum Maintenance

All experiments were performed with potted grapevine plants using the downy mildew resistant cultivars of *V. vinifera* Solaris, Cabernet Blanc and Phoenix; the susceptible cultivars Riesling, Merlot, Moscato; and the susceptible cultivar Niagara Rosada (*V. labrusca*). The cuttings were grown in pots containing sterilized substrate (manure, clay soil and sand at a ratio of 3:3:1) under greenhouse conditions where the average air temperature was 25 ± 5°C (error SEM), the RH was 60% and the photosynthetic active radiation (PAR) was 500 μmol m^–2^ s^–1^ with a 12-h photoperiod. The substrate was fertilized with 3 g of Osmocote^®^ per gram of soil. After budbreak, the plants were conducted in a single stem and top pruned when they presented 6–7 fully expanded leaves. The plants received 200 mL of water daily and were fertilized monthly with 10 g of Hakaphos^®^ Blau.

Leaves with symptoms of downy mildew were collected in vineyards, and sporangia of *P. viticola* were harvested, dried at room temperature and maintained at −25°C in Eppendorf tubes. Sporangia were rehydrated in 50 mL of bidistilled water and suspensions of the inoculum at a concentration of 10^4^ sporangia mL^–1^ were obtained using a Neubauer chamber. One month after budbreak, potted plants of cv. Merlot were inoculated by spraying inoculum suspension in the abaxial side of leaves up to runoff. Immediately after the inoculation, plants were kept in an incubation chamber at 25°C and covered with a black plastic bag to keep leaves in darkness and high air relative humidity (>90%) for 24 h. Three drops of 50 μL each of the sporangia suspension used for inoculation were placed in a polystyrene dish and kept in the same incubation chamber of inoculated plants to evaluate the viability of sporangia. Viability of sporangia was determined by the observation at light microscope of the opened sporangium 24 h after incubation. Afterward, the plastic bags were removed and plants were kept in the same incubation chamber with a 12-h photoperiod and PAR of 400 μmol m^–2^ s^–1^. The inoculum production was carried out by successive inoculations of *P. viticola* suspensions into new healthy potted plants every month.

### Gas Exchange Variables in Cultivars Resistant and Susceptible to Downy Mildew

Seven experiments were performed, one per cultivar, with the three resistant and the four susceptible cultivars. Five plants of each cultivar were inoculated as described above and kept at humid chamber for 12 h. As a control, five plants were sprayed with distilled water. The experiments were performed in the full randomized design. Net photosynthetic rate (*A*), stomatal conductance (*g*_*s*_), intercellular CO_2_ concentration (*C*_*i*_) and transpiration (*E*) were measured on the inoculation day (day 0) and 1, 4, and 12 days after the inoculation (dai) of *P. viticola* in all resistant cultivars, except for cv. Solaris in which the gas exchange was evaluated at 0, 1, 2, 5, and 12 dai. In the susceptible cultivars the gas exchange variables were measured at 0, 1, 2, 6, and 12 dai. The instantaneous carboxylation efficiency (*k*) was estimated as *A*/*C*_i_ (Machado et al., 2005) in each measurement of leaf gas exchange. The evaluations were performed with portable infrared gas analyzers (GFS3000, Heinz Walz GmbH, Germany; and Li-6400-XT, LI-COR Inc., United States) equipped with fluorometers (PAM 3055 and 6400-40, respectively). Cuvette temperature was set to 25°C and relative humidity was 60% at the inlet of the cuvette. The cuvette sizes were 2 and 4 cm^2^, respectively for Li-6400-XT and GFS3000. CO_2_ concentration was kept constant at 400 μmol mol^−1^. A LED array provided PAR of 1000 μmol m^–2^ s^–1^. *A, g*_s_, *C*_i_ and *E* were recorded after fluxes had stabilized. The leaf areas evaluated for gas exchange were photographed at 4 and 12 dai and digital images were processed with Quant software (Vale et al., 2001) to estimate downy mildew severity. All experiments, except for cv. Phoenix, were repeated once. The average values of photosynthetic variables measured in healthy and diseased plants were compared by Student’s *t*-test (*p* ≤ 0.05) using STATISTICA 6.0 software (StatSoft Inc., Tulsa, OK, United States). The normal distribution of data was verified by the Shapiro-Wilk’s test and transformations were performed when necessary (Supplementary Table S1).

### Photosynthetic and Photochemical Evaluations in Solaris and Riesling Cultivars

The photosynthetic limitations caused by *P. viticola* were evaluated in the grapevine cultivars Solaris and Riesling, which are resistant and susceptible to downy mildew, respectively. Three plants of each cultivar were inoculated, and three plants were sprayed with water as described previously. Six evaluations were performed in each experiment from the 5th day after inoculation onward (twice a day) using the GFS3000 (Heinz Walz, GmbH, Germany) equipped with a fluorometer (PAM 3055). Leaf CO_2_ assimilation response to increasing air CO_2_ concentration (*C*_a_) was initially evaluated at 400 μmol mol^–1^, which was gradually changed to 250, 200, 150, and 50 μmol mol^–1^ and then gradually increased to 400, 600, 800, 1400, and 2000 μmol mol^–1^. Day respiration (*R*_d_) was obtained through a linear regression between *A* (response variable) and *C*_i_ (predictor variable) under C_*a*_ <400 μmol mol^–1^ and corresponds to *A* when *C*_i_ is zero. Mesophyll conductance (*g*_m_) was estimated as follows (Flexas et al., 2007):

(1) g=mA/(C-i(Γ*(J+8(A+R)d))/(J-4(A+R)d))

where *Γ*^∗^ is the photosynthetic compensation point, i.e., the CO_2_ concentration at which the photorespiratory efflux of CO_2_ is equal to the CO_2_ photosynthetic assimilation rate; and *J* is the transport of electrons from chlorophyll fluorescence assessments. The calculated values of *g*_m_ were used to convert *A*-*C*_i_ curves into *A*-*C*_c_ (*C*_c_ is CO_2_ concentration at carboxylation sites in chloroplasts) curves using the following equation (Flexas et al., 2007):

(2) C=cC-iA/gm

From *A*-*C*c curves, the maximum Rubisco carboxylation rate (*V*_cmax_) and maximum rate of electron transport driving regeneration of ribulose-1,5-bisphosphate (*J*_max_) were estimated as follows (Farquhar et al., 1980; Flexas et al., 2007):

(3) A=V(C-cΓ*)cmax/(C+cK((1-(O/K)o))c

(4) A=J(C-cΓ*)max/4(C+c2Γ*)

where *K*_c_ and *K*_o_ are the Michaelis-Menten constants of Rubisco for carboxylation and oxygenation (Bernacchi et al., 2003), respectively, and *O* is the internal O_2_ concentration, which is considered equal to the external O_2_ concentration. *V*_cmax_ and *J*_max_ were estimated by non-linear regressions using STATISTICA 6.0 software (StatSoft Inc., Tulsa, OK, United States).

The maximum leaf CO_2_ assimilation (*A*_max_) and the maximum stomatal conductance (*g*_smax_) were also obtained in each *A*-*C*_c_ curve. The experiment was performed twice and the average values of photosynthetic variables measured in healthy and diseased plants were compared using Student’s *t*-test (*p* ≤ 0.05).

### Detection and Quantification of Chlorophyll *a* Fluorescence, H_2_O_2_, O_2_^–^ and APX Activity

Chlorophyll *a* fluorescence was measured using an Imaging-PAM Maxi (Heinz Walz GmbH, Germany) in cv. Solaris. The measurements were performed 6, 12, 30, 60, 120, and 288 h after *P. viticola* inoculation (hai). After dark-adapting leaves for 20 min, a saturation light pulse of 2700 μmol m^–2^s^–1^ was applied and the maximum PSII quantum yield [F_v_/F_m_ = (F_m_−F_o_)/F_m_] was measured (Maxwell and Johnson, 2000). F_m_ and F_o_ denote the maximum and minimum fluorescence of dark-adapted samples, respectively.

The detection of H_2_O_2_ and O_2_^–^ was performed in cvs. Solaris and Riesling and its quantification was only performed where H_2_O_2_ was detected. The samples were collected at 6, 12, 30, 60, 120, and 288 hai. For quantification of H_2_O_2_, five leaf discs of 0.46 cm^2^ were removed with a cork borer approximately 2 cm away from the central vein of healthy and inoculated leaves. All five discs were immediately transferred to 1 mL of acetone acidified with 25 mM H_2_SO_4_ and frozen in liquid nitrogen, where they remained until measurements. For measurements, 50 μL of thawed samples were added to 1 mL of ferrous ammonium sulfate xylenol orange (FOX) solution that contained 250 μM ferrous ammonium sulfate, 100 mM sorbitol, 100 μM xylenol orange and 25 mM H_2_SO_4_. The samples were then incubated at room temperature for 30 to 45 min. H_2_O_2_ concentration was determined at 550 nm (EPOCH, BioTec, United States) and 850 nm (8453 UV–vis Spectroscopy System, Agilent, United States) using a standard curve ranging from 0 to 100 μM H_2_O_2_ (Wolff, 1994).

Leaf samples (0.5 g) were harvested and immediately frozen in liquid nitrogen. To assess ascorbate peroxidase (APX) activity, samples were homogenized in 5 mL phosphate buffer (pH 7.6) including 1% polyvinylpyrrolidone (PVP) and 0.1 mM EDTA and centrifuged for 20 min at 16,000 *g* at 4°C. The supernatant was collected and used as crude extract in the reaction mixtures of the enzyme activity assays. For the APX assay, the 0.3-mL reaction mixture contained 0.5 mM ascorbic acid, 50 mM phosphate buffer, 1 mM EDTA, 0.5 mM H_2_O and 10–15 μL of the supernatant. The reaction was started by adding 10 μL of 15 mM of H_2_O_2_, and APX was assayed spectrometrically (EPOCH, BioTec, United States/8453 UV-VIS Spectroscopy System, Agilent, United States) following the decrease in absorbance at 290 nm (Nakano and Asada, 1981).

Histochemical detection of H_2_O_2_ and O_2_^–^ was performed on leaf discs (3 cm^2^) removed from the same leaves used for the estimation of H_2_O_2_ concentration. The samples were collected at 6, 12, 30, 60, 120, and 288 hai from each plant of each treatment. The samples were placed in glass vials containing 3,3′−diaminobenzidine tetrahydrochloride (DAB), pH 5.5, 1%; Sigma−Aldrich, or nitroblue tetrazolium (NBT), pH 6, 0.1%; Sigma−Aldrich, and kept at 25°C for 2 h in darkness (Thordal-Christensen et al., 1997). After this period, the samples were cleared in 80% ethanol for 24 h, stored in glycerol solution (70%), and then observed with an optical microscope. The average values of H_2_O_2_ concentrations measured in healthy and diseased plants were compared by Student’s *t*-test (*p* ≤ 0.05).

## Results

### Differential Gas Exchange Responses of Resistant and Susceptible Grapevine Cultivars to *Plasmopara viticola* Infection

The death-like hypersensitive reactions “HR” were observed 4 dai with *P. viticola* on the resistant cultivars Solaris, Cabernet Blanc and Phoenix (Supplementary Figure S1). In the susceptible cultivars, the symptoms of downy mildew and *P. viticola* sporulation were observed 6 to 7 dai. The visual area affected by “HR” did not change for cvs. Cabernet Blanc and Phoenix after 4 dai, and a slow progression (from 5.9% at 4 dai to 10.2% at 12 dai) was exclusively noticed in cv. Solaris. After 12 dai, necrotic areas accounted for 2.3 and 3.3% (on average) of Cabernet Blanc and Phoenix, respectively (Supplementary Figures S1, S2). Typical symptoms of downy mildew, i.e., oil spots on the adaxial leaf surface and abundant sporulation of *P. viticola* on the abaxial leaf surface (Supplementary Figure S2), were observed on all susceptible cultivars. Disease severity reached 47, 53, 46, and 34% in Riesling, Niagara Rosada, Merlot and Moscato, respectively.

**FIGURE 1:**
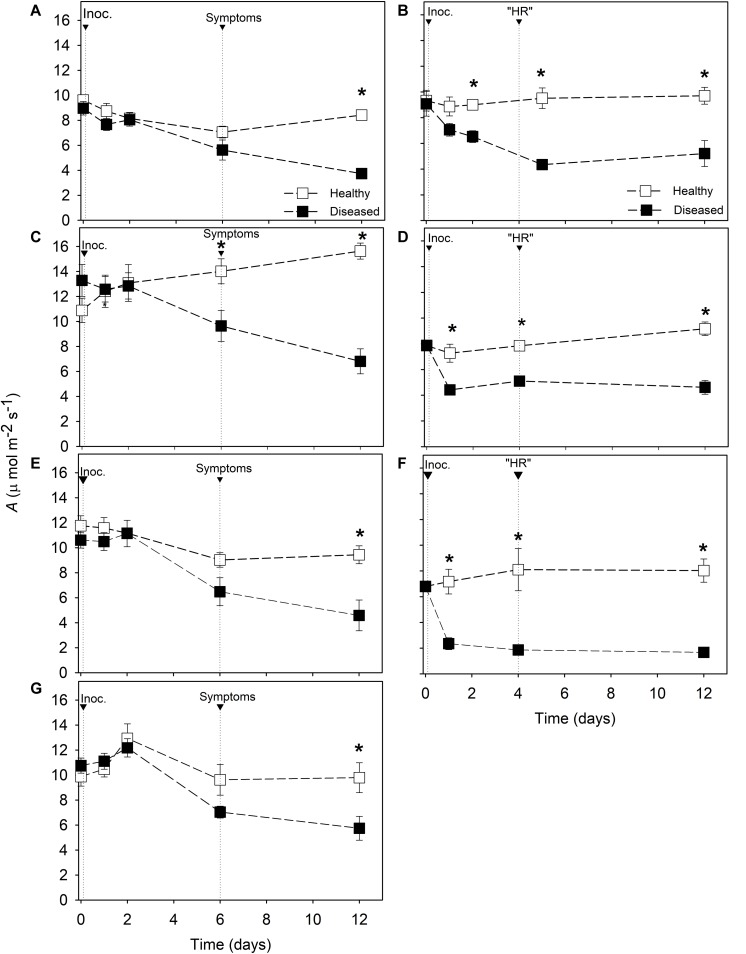
Net photosynthetic rate *(A)* in *Vitis* spp. cultivars. Downy mildew symptoms were observed in Riesling **(A)**, Niagara Rosada **(C)**, Merlot **(E)** and Moscato **(G)** 6 days after inoculation. Cell death-like hypersensitive reactions “HR” were observed in Solaris **(B)**, Cabernet Blanc **(D)** and Phoenix **(F)** 4 days after inoculation (Inoc.). The average values of healthy and diseased leaves were compared using the Student’s *t* test for each cultivar (*n* = 6, ± SE), and * indicates significant differences (*p* < 0.05).

On the day of inoculation with *P. viticola*, photosynthetic rates were similar for inoculated and non-inoculated leaves of resistant cultivars Solaris, Cabernet Blanc and Phoenix (Figure 1). However, significant reductions of leaf photosynthesis (*A*) were found at 2 dai in Solaris (Figure 1B) and at 1 dai for Cabernet Blanc and Phoenix (Figures 1D,F). At the appearance of “HR” and compared with healthy leaves, *A* values were 54, 35, and 77% lower on diseased leaves of Solaris, Cabernet Blanc, and Phoenix, respectively (Figures 1B,D,F). Then, the *A* values for inoculated leaves did not change significantly until 12 dai.

Photosynthetic rates ranged from 8.9 to 13.3 μmol m^–2^ s^–1^ on susceptible cultivars at the day of inoculation when no differences were found between inoculated and non-inoculated leaves (Figures 1A,C,E,G). When visual symptoms appeared, significant reduction of *A* was only observed in Niagara Rosada (Figure 1C). After 12 days of inoculation, *A* values on inoculated leaves were reduced by 56, 56, 51, and 41% compared with healthy leaves of Riesling, Niagara Rosada, Merlot, and Moscato, respectively.

**FIGURE 2:**
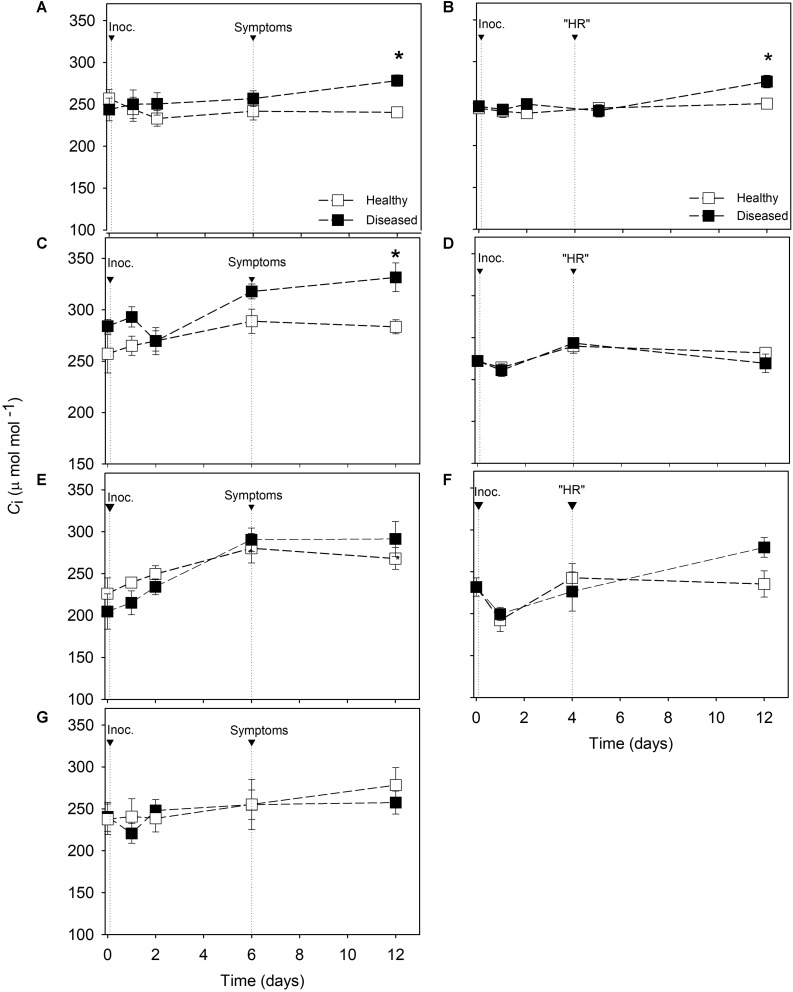
Intercellular CO_2_ concentration (*C*_i_) in *Vitis* spp. cultivars. Downy mildew symptoms were observed in Riesling **(A)**, Niagara Rosada **(C)**, Merlot **(E)** and Moscato **(G)** 6 days after inoculation. Cell death-like hypersensitive reactions “HR” were observed in Solaris **(B)**, Cabernet Blanc **(D)** and Phoenix **(F)** 4 days after inoculation (Inoc.). The average values of healthy and diseased leaves were compared using the Student’s *t* test for each cultivar (*n* = 6, ± SE), and * indicates significant differences (*p* < 0.05).

No differences in *C*_i_ of inoculated and non-inoculated leaves were found before the appearance of “HR” in resistant cultivars (Figure 2), and this situation persisted until 12 dai for Cabernet Blanc and Phoenix. A slight increase (10.5%) was detected on *C*_*i*_ of inoculated leaves of Solaris 12 dai compared with non-inoculated leaves (Figure 2B). Similar *C*_*i*_ values were measured on inoculated and non-inoculated leaves of susceptible cultivars until symptoms appearance (Figures 2A,C,E,G). From this time, diseased leaves of Riesling and Niagara Rosada showed increases in *C*_*i*_ values of 14% compared with healthy leaves (Figures 2A,C). Similar to Cabernet Blanc and Phoenix (Figures 2D,F), *C*_*i*_ of Merlot and Moscato remained stable from 6 to 12 dai regardless of the treatment (Figures 2E,G).

**FIGURE 3:**
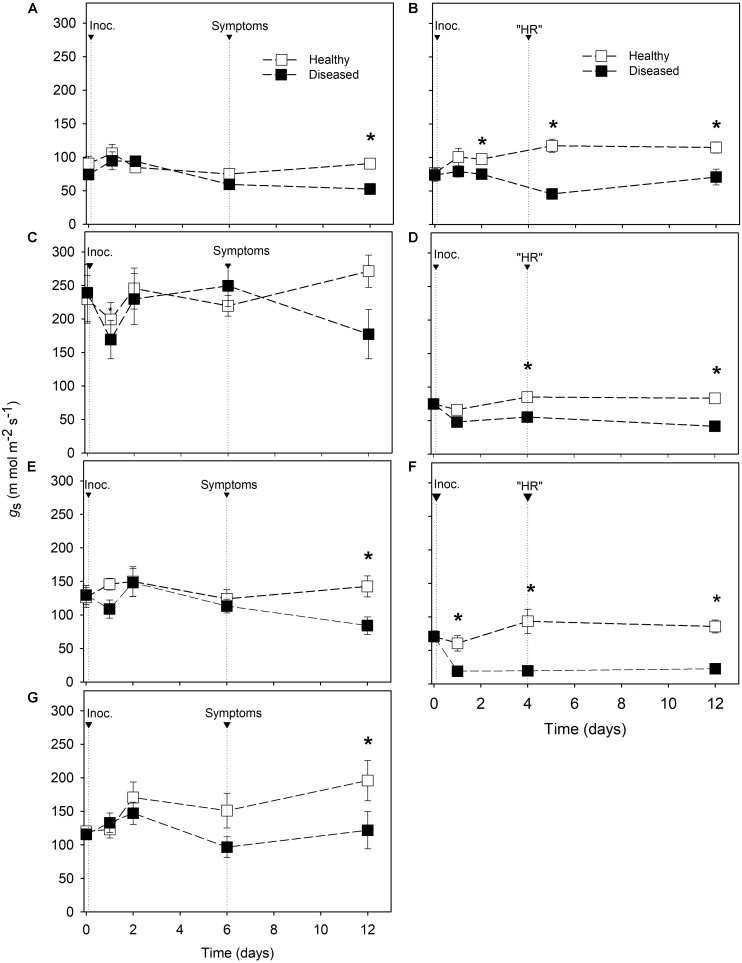
Stomatal conductance (*g*_*s*_) in *Vitis* spp. cultivars. Downy mildew symptoms were observed in Riesling **(A)**, Niagara Rosada **(C)**, Merlot **(E)** and Moscato **(G)** 6 days after inoculation. Cell death-like hypersensitive reactions “HR” were observed in Solaris **(B)**, Cabernet Blanc **(D)** and Phoenix **(F)** 4 days after inoculation (Inoc.). The average values of healthy and diseased leaves were compared using the Student’s *t* test for each cultivar (*n* = 6, ± SE), and ^∗^ indicates significant differences (*p* < 0.05).

**FIGURE 4:**
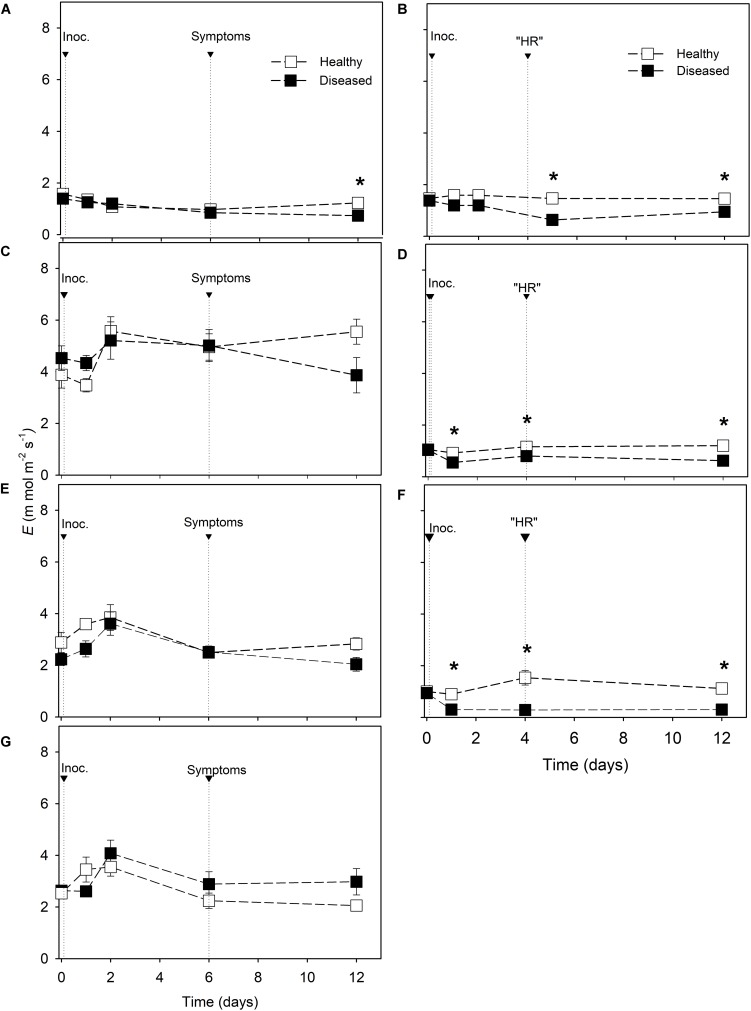
Transpiration rate (*E*) in *Vitis* spp. cultivars. Downy mildew symptoms were observed in Riesling **(A)**, Niagara Rosada **(C)**, Merlot **(E)** and Moscato **(G)** 6 days after inoculation. Cell death-like hypersensitive reactions “HR” were observed in Solaris **(B)**, Cabernet Blanc **(D)** and Phoenix **(F)** 4 days after inoculation (Inoc.). The average values of healthy and diseased leaves were compared using the Student’s *t* test for each cultivar (*n* = 6, ± SE), and * indicates significant differences (*p* < 0.05).

Considering downy mildew resistant cultivars, *g*_*s*_ and *E* were always lower in diseased leaves than in healthy leaves from 4 dai (Figures 3, 4). Differences in *g*_*s*_ between inoculated and non-inoculated leaves of susceptible cultivars were observed only at 12 dai in Riesling, Merlot, and Moscato (Figures 3A,E,G). Nevertheless, no reductions in *E* were found in susceptible cultivars with the exception of Riesling at 12 dai when the average *E* value of diseased leaves was reduced by 40% compared with healthy leaves (Figure 4A).

Significant reductions in the instantaneous carboxylation efficiency (*k*) due to downy mildew were measured from 1 dai for resistant cultivars and at 12 dai for susceptible cultivars (Supplementary Figure S3). In Niagara Rosada, reduction in *k* was also observed in diseased leaves at 6 dai (Supplementary Figure S3C).

### Photosynthetic and Photochemical Evaluations in Solaris and Riesling Cultivars

**FIGURE 5:**
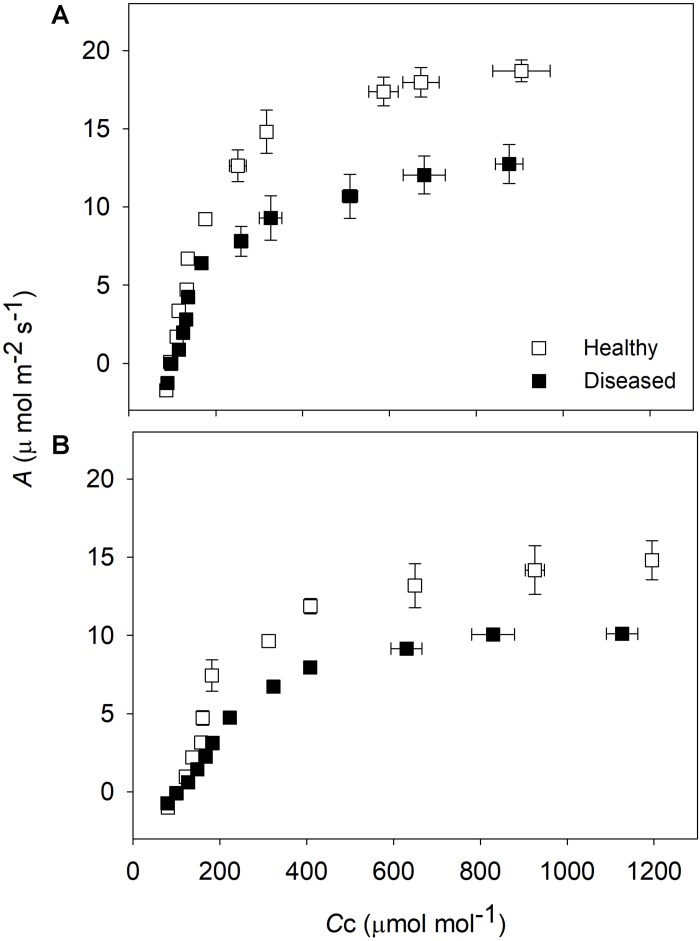
Response of leaf CO_2_ assimilation (*A*) to increasing CO_2_ concentration at carboxylation sites in chloroplasts (*C*_*c*_) of *Vitis vinifera* cv. Solaris **(A)** and Riesling **(B)**. Open symbols represent mean values (*n* = 3, ± SE) of healthy leaves, while filled symbols indicate diseased leaves.

Healthy plants were more responsive to increasing chloroplastic CO_2_ concentrations when compared with *P. viticola* inoculated plants regardless of the cultivar (Figure 5). The maximum leaf CO_2_ assimilation (*A*_*max*_) was 18.7 and 12.7 μmol m^–2^ s^–1^ in healthy and diseased leaves of Solaris, respectively, and a reduction of 23% was noticed due to *P. viticola* infection. *V*_*c*__*max*_ was similar in healthy and diseased leaves of Solaris; however, *J*_*max*_ was reduced by 34% in diseased leaves (Table 1). The maximum stomatal conductance (*g*_*smax*_) was reduced (−27%) in Solaris without any significant change in *g*_*m*_ (Table 1). In Riesling, *A*_*max*_ was 22% higher in healthy leaves than in diseased leaves. Unlike Solaris, biochemical limitations were higher in diseased leaves of Riesling, with plants showing low *V*_*c*__*max*_ and *J*_*max*_ (Table 1). In addition, no significant diffusive limitation was found in diseased leaves compared with healthy leaves of Riesling, as suggested by *g*_*m*_ and *g*_*smax*_ (Table 1).

**TABLE 1:** Maximum Rubisco carboxylation rate (*V*_*cmax*_), maximum rate of electron transport driving regeneration of ribulose-1,5-bisphosphate (*J*_*max*_), mesophyll conductance (*g*_*m*_), maximum stomatal conductance (*g*_*smax*_), and maximum photosynthetic rate (*A*_*max*_) in Solaris and Riesling cultivars as affected by downy mildew (*Plasmopara viticola*).

Variable	Healthy plants	Infected plants	*t*-test
**Solaris**			
*V*_*cmax*_ (μmol m^–2^ s^–1^)	83.69.6	70.511.1	ns
*J*_*max*_ (μmol m^–2^ s^–1^)	131.29.2	86.47.7	*
*g*_*m*_ (mol m^–2^ s^–1^)	0.110.03	0.050.02	ns
*g*_*smax*_ (mmol m^–2^ s^–1^)	143.56.5	105.15.6	*
*A*_*max*_ (μmol m^–2^ s^–1^)	18.70.7	12.71.3	*
**Riesling**			
*V*_*cmax*_ (μmol m^–2^ s^–1^)	58.45.9	33.00.8	*
*J*_*max*_ (μmol m^–2^ s^–1^)	88.15.5	65.70.2	*
*g*_*m*_ (mol m^–2^ s^–1^)	0.100.02	0.080.01	ns
*g*_*smax*_ (mmol m^–2^ s^–1^)	117.212.6	90.52.4	ns
*A*_*max*_ (μmol m^–2^ s^–1^)	13.10.6	10.10.2	*

For *t*-test, * means statistical difference at *p* ≤ 0.05 whereas ns means non-significant difference. Mean values (*n* = 3) ± SE.

**FIGURE 6:**
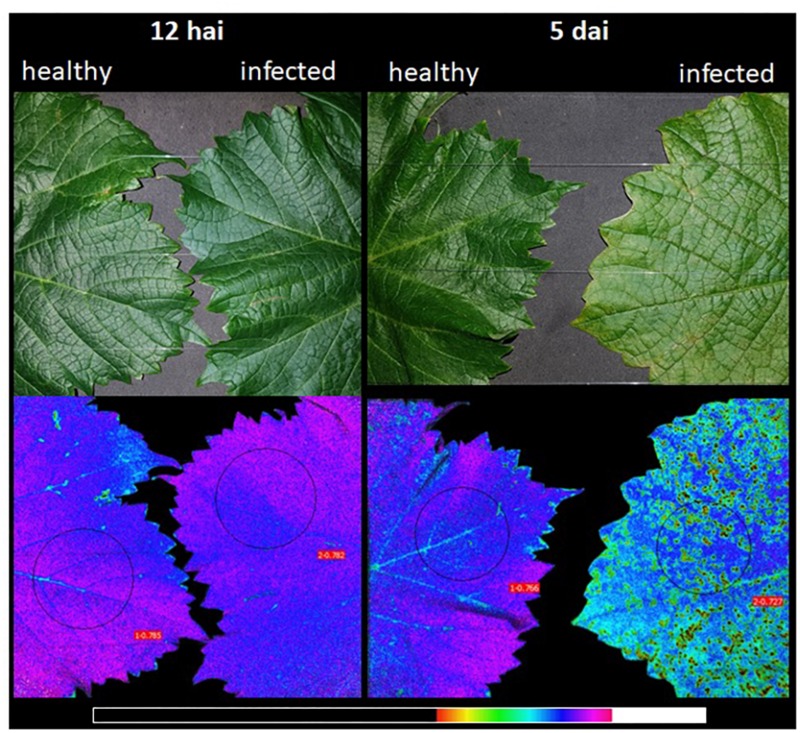
Digital and chlorophyll fluorescence images of grapevine leaves of cv. Solaris after 12 h and 5 days after infection (dai) with *Plasmopara viticola*. Chlorophyll fluorescence images show the maximum PSII quantum efficiency (F_*v*_/F_*m*_). Circles indicate the area used for calculation of F_*v*_/F_*m*_. Leaves were dark-adapted prior to measurements. The false color code depicted at the bottom of images represents the range of 0 (black) to 1 (white).

Chlorophyll fluorescence from healthy and inoculated leaves of Solaris indicated similar maximum PSII quantum efficiency (F_*v*_/F_*m*_) at 12 hai (0.770 and 0.766). Reduced F_*v*_/F_*m*_ in inoculated leaves was found at 5 dai (0.735) when “HR” symptoms were visible (Figure 6). In non-inoculated leaves, F_*v*_/F_*m*_ was 0.752. Although the effects of *P. viticola* infection are clearly visible by imaging of chlorophyll *a* fluorescence, no significant difference was detected on any day (Supplementary Figure S4).

### Oxidative Burst in the Resistant Cultivar Solaris

**FIGURE 7:**
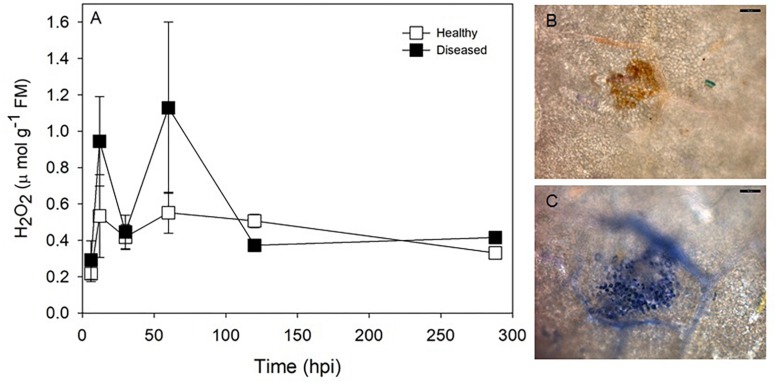
Time-course of leaf H_2_O_2_ concentration **(A)** in healthy and *P. viticola* infected leaves of cv. Solaris after inoculation and visual localization of H_2_O_2_ **(B)** and O_2_^–^ **(C)** in *P. viticola* inoculated leaves, 12 h after inoculation. Scale bar = 50 μm.

The presence of H_2_O_2_ as indicated by DAB staining was detected as brown spots close to the secondary veins of diseased leaves (Figure 7B). Brown spots were frequent and intense at 12 and 30 hai (Supplementary Figure S5). Necrotic tissue formed on diseased leaves from 120 hai, and no reaction to DAB staining was observed in diseased leaves at this time (data not shown). Brown spots were not detected in healthy leaves. H_2_O_2_ concentrations were lower than 0.5 μmol g^–1^ fresh mass (FM) in both healthy and diseased leaves of Solaris at 6 hai and from 120 hai. However, increases in leaf H_2_O_2_ concentration and data variability were noticed at 12 and 60 hai (Figure 7). O_2_**^–^** was detected as dark blue spots similar in shape to those spots observed in H_2_O_2_ detection and near secondary veins. Similar to H_2_O_2_, the frequency and intensity of blue spots were high at 12 hai (Figure 7C). No blue spots were observed at 30 hai in diseased leaves, and no O_2_**^–^** was detected in healthy leaves (data not shown). No significant difference was observed for APX activity between healthy and diseased leaves, although the highest APX activity (2.33 μmol H_2_O_2_ g^–1^ FM min^–1^) was noticed in diseased leaves at 288 hai (Supplementary Figure S6).

## Discussion

The results of this study indicate that *P. viticola* infection is associated with a cost in terms of CO_2_ assimilation per unit of leaf area, i.e., resistant cultivars reduced *A* earlier and more severely than susceptible cultivars. On average, 55% reduction in leaf CO_2_ assimilation was noticed few hours after pathogen contact with its host, and such a decrease remained up to 12 days after *P. viticola* inoculation in resistant cultivars Solaris, Cabernet Blanc and Phoenix. This behavior could result from the downregulation of proteins related to photosynthesis and carbohydrate metabolism as previously reported in resistant grapevine cultivars (Figueiredo et al., 2017; Nascimento-Gavioli et al., 2017). A fast and massive transcription of signaling genes affected by *P. viticola* has also been reported on *Rpv*-bearing in grapevine cultivars (Fröbel et al., 2019). This response presumably is initiated by a receptor gene that triggers the signal transduction cascade such as that noted in a pathogen-associated molecular pattern (PAMP, Fröbel et al., 2019). In contrast, no effect on leaf gas exchange was observed during the infection and colonization of *P. viticola* in susceptible cultivars. The net photosynthetic rate on susceptible cultivars was reduced only when symptoms appeared at approximately 6 days after pathogen inoculation. In the susceptible cultivar Trincadeira there was no reduction of photosynthetic pigments content 24 h after *P. viticola* inoculation (Nascimento et al., 2019). This suggests that the photosynthetic rate remains unaltered on the first stages of the interaction where there are no visible lesions or symptoms (Nascimento et al., 2019). Similarly to our results, in the biotroph interaction between barley and powdery mildew (*Blumeria graminis*), no reduction of photosynthesis was observed in compatible interactions up to 3 dai. However, the photosynthesis in those leaves was progressively reduced up to 60% compared with healthy leaves at 7 dai (Swarbrick et al., 2006).

*Plasmopara viticola* infection increased stomatal closure and decreased transpiration rates of resistant cultivars from 4 dai. Stomatal closure is a mechanism related to grapevine resistance to downy mildew, impairing pathogen penetration and sporulation (Allègre et al., 2009). As soon as *P. viticola* zoospores are released from sporangia, they move toward stomata by chemotaxis, encyst and rapidly penetrate leaf tissue through the stomata in susceptible cultivars. Partial stomatal closure contributes to avoiding *P. viticola* penetration in resistant grapevine cultivars, although it is insufficient to limit infection (Allègre et al., 2009). Stomatal conductance was reduced only 12 days after inoculation in most susceptible cultivars and was less affected when compared with photosynthesis. In cv. Marselan, a susceptible cultivar to downy mildew, *P. viticola* affects guard cell function and interferes with stomatal opening, as observed for other oomycetes (Allègre et al., 2007). For instance, *Phytophthora infestans* can cause abnormal stomata functioning in potato leaves to facilitate the emergence of sporangiophores and sporulation in dark conditions (Farrell et al., 1969).

Although drastic alterations were observed in *A*, *g*_*s*_ and *E* of inoculated resistant grapevines, no variation was noticed in *C*_*i*_ of Cabernet Blanc and Phoenix leaves while it increased in inoculated leaves of Solaris at 12 dai. This was somehow unexpected since decreases in *C*_*i*_ are expected in leaves due to decreases in stomatal conductance. However, the low variation in *C*_*i*_ of resistant cultivars and the reduction in the instantaneous carboxylation efficiency after the infection (Supplementary Figure S3) in parallel to stomatal closure suggest that another physiological process controls intercellular CO_2_ concentrations of *P. viticola*-infected leaves. A rapid increase in the respiration rate of barley leaves resistant to *Erysiphe graminis* was reported after pathogen contact, while such increase in respiration was found only 3 days after inoculation in compatible interactions (Smedegaard-Petersen and Tolstrup, 1985). A late increase in *C*_*i*_ after the development of disease symptoms was observed in downy mildew-susceptible cultivars, which is in agreement with the linear increase in *C*_*i*_ with increasing rust severity observed in *V. labrusca* infected with *Phakopsora euvitis* (Nogueira Júnior et al., 2017).

The activation of signal transduction pathways following the recognition of *P. viticola* by resistant grapevines leads to the production of reactive oxygen species (Aziz et al., 2003), such as O_2_^–^ and H_2_O_2_ (Kortekamp and Zyprian, 2003). H_2_O_2_ production is one of the earliest detectable cytological responses to downy mildew in resistant cultivars, as noticed in Solaris 12 h after inoculation (our data and Trouvelot et al., 2008). The production of reactive oxygen species is associated with localized cell death and is crucial for delimiting the growth of pathogen into leaf tissues (Aziz et al., 2003). Reactive oxygen species cause oxidative damage to a variety of macromolecular targets, such as DNA, lipids and proteins, including enzymes of the Calvin–Benson Cycle and proteins located in the thylakoid membrane, leading to irreversible damage and ultimately tissue necrosis (Halliwell, 2006). Oxidative stress blocks key steps in chlorophyll biosynthesis by directly or indirectly inhibiting the enzymes involved in chlorophyll synthesis (Aarti et al., 2006). Herein, necrotic areas were observed 4 days after inoculation and ranged from 1.8 to 6.0% of total leaf area in resistant cultivars Solaris, Cabernet Blanc and Phoenix. Solaris inoculated with *P. viticola* showed low maximum PSII quantum efficiency 5 days after inoculation and low regeneration of RuBP driven by electron transport (Figure 6 and Table 1). This photochemical limitation is presumably related to the production of H_2_O_2_ and the degradation of cell photosynthetic pigments. As shown in Figure 6, the occurrence of distinct spots indicative of low photosynthetic efficiency is clearly visible upon infection with *P. viticola*. These spots might contribute to overall reduction on photosynthetic performance. In grapevine leaves of cv. Marselan infected with *P. viticola*, chlorophyll concentrations were reduced by approximately 30% (Gamm et al., 2011). Measurements conducted in spots and adjacent areas without symptoms confirmed that F_*v*_/F_*m*_ was reduced in the spots, but the asymptomatic adjacent areas had F_*v*_/F_*m*_ values comparable to non-infected leaves (Gamm et al., 2011). In the present study, F_*v*_/F_*m*_ tends to decrease at 5 dai (although not statistically significant). This might be due to the occurrence of necrotic spots that are present in the leaf area considered for calculation of F_*v*_/F_*m*_. Reduced F_*v*_/F_*m*_ is an indicator for impaired PSII functionality due to damage to reaction center proteins by reactive oxygen species, and this finding is consistent with reduced assimilation rates. In susceptible cultivars, such as Riesling (this study), Sangiovese (Moriondo et al., 2005) and Niagara Rosada (Nogueira Júnior et al., 2019), the increase in photochemical limitations is also observed during downy mildew symptom expression. Low concentrations of photosynthetic pigments in the yellow halos surrounding *P. viticola* lesions are related with the reduction in photochemical reactions in these cultivars (Moriondo et al., 2005).

Expression of plant defenses is often assumed to be costly, requiring diversion of resources away from plant growth and development (Walters and Boyle, 2005), and *P. viticola* greatly enhances carbohydrate hydrolysis and represses photosynthesis-associated proteins in resistant cultivars (Nascimento-Gavioli et al., 2017). In the incompatible interaction of barley and *Erysiphe graminis* f. sp. *hordei*, pathogen recognition occurs during haustoria development into host cells. After recognition, several histochemical and biochemical alterations are induced in hosts (Smedegaard-Petersen and Tolstrup, 1985). The cost to the net photosynthetic rate in barley leaves challenged by *E. graminis* f. sp. *hordei* is also similar to the cost observed in downy mildew-resistant grapevines. However, in the incompatible interaction of *Eucalyptus grandis* and *Puccinia psidii*, the host reacts immediately during pathogen appressoria formation. Consequently, only small flecks are observed in the host before pathogen penetration and photosynthetic activity is unaffected (Alves et al., 2011).

Although resistant cultivars decrease the photosynthetic activity when infected by *P. viticola*, its use provides a substantial contribution to the sustainability of viticulture while reducing pesticide applications (Buonassisi et al., 2017). *P. viticola* can complete its life cycle in resistant cultivars. However, low oomycete sporulation is noted compared with the sporulation in susceptible cultivars, and sporangiophores are abnormal. The reduced sporulation affects the secondary spread of the pathogen and reduces the rate of disease progression in field (Parlevliet, 1979). In the last 20 years, newly bred grapevine cultivars with disease resistance, desirable agronomic attributes and good enological characteristics have been introduced to the market (Buonassisi et al., 2017). These cultivars bear different *Rpv* resistance loci to downy mildew and several studies have been developed to identify the resistance loci on these materials. Locus *Rpv 3* is present in all resistant cultivars used in the present work. However, the cultivar Solaris has also the *Rpv10* locus^1^. In general, the effect of *P. viticola* on photosynthesis was similar for cvs. Solaris, Cabernet Blanc and Phoenix.

## Conclusion

In conclusion, *P. viticola* differently affects leaf gas exchange in resistant and susceptible cultivars, with resistant cultivars exhibiting faster responses. While net photosynthetic rate and stomatal conductance of resistant cultivars are rapidly and negatively affected by *P. viticola*, susceptible cultivars only exhibit reduced leaf gas exchange at late stages of disease development. These results would suggest that the recognition followed by induced resistance responses is costly for grapevine carbon assimilation. In the absence of recognition, the pathogen colonizes the leaf tissue without affecting photosynthesis until the appearance of visual symptoms. Although the induction of defenses in resistant grapevine cultivars is costly, the use of genetic resistance to control downy mildew as an alternative to chemical control is highly desirable and complementary for a sustainable viticulture.

## Data Availability Statement

The raw data supporting the conclusions of this article will be made available by the authors, without undue reservation, to any qualified researcher.

## Author Contributions

AN and LA devised the project and the main conceptual ideas. AN and MT performed the experiments. AN, LA, RR, and AT revised critically the project and the manuscript for important intellectual content. All authors discussed the results and contributed to the final manuscript.

## Conflict of Interest

The authors declare that the research was conducted in the absence of any commercial or financial relationships that could be construed as a potential conflict of interest.

## Footnotes

1 www.vivc.de

## References

[B1] Aarti P. D. Tanaka R. Tanaka A. (2006). Effects of oxidative stress on chlorophyll biosynthesis in cucumber (*Cucumis sativus*) cotyledons. *Physiol Plant.* 128 186–197. 10.1111/j.1399-3054.2006.00720.x

[B2] Agrios G. N. (2005). *Plant Pathology*, fifth Edn San Diego: Elsevier Academic Press.

[B3] Allègre M. Daire X. Héloir M. C. Trouvelot S. Mercier L. Adrian M. (2007). Stomatal deregulation in *Plasmopara viticola* – Infected grapevines leaves. *New Phytol.* 173 832–840. 10.1111/j.1469-8137.2006.01959.x 17286831

[B4] Allègre M. Héloir M. C. Trouvelot S. Daire X. Pugin A. Wendehenne D. (2009). Are grapevine stomata involved in the elicitor-induced protection against downy mildew? *Mol. Plant Microbe Interact.* 22 977–986. 10.1094/mpmi-22-8-0977 19589073

[B5] Alves A. A. Guimarães L. M. S. Chaves A. R. M. C. DaMatta F. M. Alfenas A. C. (2011). Leaf gas exchange and chlorophyll a fluorescence of *Eucalyptus urophylla* in response to *Puccinia psidii* infection. *Acta Physiol. Plant.* 33 1831–1839. 10.1007/s11738-011-0722-z

[B6] Armijo G. Schlechter R. Agurto M. Muñoz D. Nuñez C. Arce-Johnson P. (2016). Grapevine pathogenic microorganisms: understanding infection strategies and host response scenarios. *Front. Plant Sci.* 7:382. 10.3389/fpls.2016.00382 27066032 PMC4811896

[B7] Ash G. (2000). Downy mildew of grape. *The Plant Health Instructor.* Available online at: http://www.apsnet.org/edcenter/intropp/lessons/fungi/Oomycetes/Pages/DownyMildewGrape.aspx (accessed November 20, 2018).

[B8] Aziz A. Poinssot B. Daire X. Adrian M. Bézier A. Lambert B. (2003). Laminarin elicits defense responses in grapevine and induces protection against *Botrytis cinerea* and *Plasmopara viticola*. *Mol. Plant Microbe Interact.* 16 1118–1128. 10.1094/MPMI.2003.16.12.1118 14651345

[B9] Bellin D. Peressotti E. Merdinoglu D. Wiedemann-Merdinoglu S. Adam-Blondon A. F. Cipriani G. (2009). Resistance to *Plasmopara viticola* in grapevine ‘Bianca’ is controlled by a major dominant gene causing localised necrosis at the infection site. *Theor. Appl. Genet.* 120 163–176. 10.1007/s00122-009-1167-2 19821064

[B10] Berger S. Sinha A. K. Roitsch T. (2007). Plant physiology meets phytopathology: plant primary metabolism and plant-pathogen interactions. *J. Exp. Bot.* 58 4019–4026. 10.1093/jxb/erm298 18182420

[B11] Bernacchi C. J. Pimentel C. Long S. P. (2003). *In vivo* temperature response functions of parameters required to model RuBP-limited photosynthesis. *Plant Cell Environ.* 26 1419–1430. 10.1046/j.0016-8025.2003.01050.x

[B12] Blasi P. Blanc S. Wiedemann-Merdinoglu S. Prado E. Rühl E. H. Mestre P. (2011). Construction of a reference linkage map of *Vitis amurensis* and genetic mapping of *Rpv8*, a locus conferring resistance to grapevine downy mildew. *Theor. Appl. Genet.* 123 43–53. 10.1007/s00122-011-1565-0 21404060

[B13] Buonassisi D. Colombo M. Migliaro D. Dolzani C. Peressotti E. Mizzotti C. (2017). Breeding for grapevine downy mildew resistance: a review of “omics” approaches. *Euphytica* 213:103 10.1007/s10681-017-1882-8

[B14] Cappello F. P. Spósito M. B. Osaki M. (2017). Production costs and profitability of ‘Niagara Rosada’ table grape grown in different regions of São Paulo state. *Rev. Bras. Frutic.* 39 e–774. 10.1590/0100-29452017774

[B15] Cipollini D. (1998). Induced defenses and phenotypic plasticity. *Trends Ecol. Evol.* 13 200 10.1016/S0169-5347(98)01366-4 21238264

[B16] Dai G. H. Andary C. Mondolot-Cosson L. Boubals D. (1995). Histochemical studies on the interaction between three species of grapevine, *Vitis vinifera*, *V. rupestris* and *V. rotundifolia* and the downy mildew fungus, *Plasmopara viticola*. *Physiol. Mol. Plant Pathol.* 46 177–188. 10.1006/pmpp.1995.1014

[B17] DeWitt T. J. Sih A. Wilson S. D. (1998). Costs and limits of phenotypic plasticity. *Trends Ecol. Evol.* 13 77–81. 10.1016/S0169-5347(97)01274-3 21238209

[B18] Díez-Navajas A. M. Wiedemann-Merdinoglu S. Greif C. Merdinoglu D. (2008). Nonhost versus host resistance to the grapevine downy mildew, *Plasmopara viticola*, studied at the tissue level. *Phytopathology* 98 776–780. 10.1094/PHYTO-98-7-0776 18943253

[B19] Farquhar G. D. von Caemmerer S. Berry J. A. (1980). A biochemical model of photosynthetic CO_2_ assimilation in leaves of C_3_ species. *Planta* 149 78–90. 10.1007/BF00386231 24306196

[B20] Farrell G. M. Preece T. F. Wren M. J. (1969). Effects of infection by *Phytophthora infestans* (Mont.) de Bary on the stomata of potato leaves. *Ann. Appl. Biol.* 63 265–275. 10.1111/j.1744-7348.1969.tb05488.x

[B21] Figueiredo A. Fortes A. M. Ferreira S. Sebastiana M. Choi Y. H. Souza L. (2008). Transcriptional and metabolic profiling of grape (*Vitis vinifera* L.) leaves unravel possible innate resistance against pathogenic fungi. *J. Exp. Bot*. 59 3371–3381. 10.1093/jxb/ern187 18648103

[B22] Figueiredo A. Martins J. Sebastiana M. Guerreiro A. Silva A. Matos A. R. (2017). Specific adjustments in grapevine leaf proteome discriminating resistant and susceptible grapevine genotypes to *Plasmopara viticola*. *J. Proteomics* 152 48–57. 10.1016/j.jprot.2016.10.012 27989945

[B23] Fischer B. M. Salakhutdinov I. Akkurt M. Eibach R. Edwards K. J. Töpfer R. (2004). Quantitative trait locus analysis of fungal disease resistance factors on a molecular map of grapevine. *Theor. Appl. Genet*. 108 501–515. 10.1007/s00122-003-1445-3 14574452

[B24] Flexas J. Diaz-Espejo A. Galmés J. Kaldenhoff R. Medrano H. Ribas-Carbo M. (2007). Rapid variations of mesophyll conductance in response to changes in CO_2_ concentration around leaves. *Plant Cell Environ.* 30 1284–1298. 10.1111/j.1365-3040.2007.01700.x 17727418

[B25] Fröbel S. Dudenhöffer J. Töpfer R. Zyprian E. (2019). Transcriptome analysis of early downy mildew (*Plasmopara viticola*) defense in grapevine carrying the Asian resistance locus *Rpv10*. *Euphytica* 215:28 10.1007/s10681-019-2355-z

[B26] Gamm M. Héloir M. C. Bligny R. Vaillant-Gaveau N. Trouvelot S. Alcaraz G. (2011). Changes in carbohydrate metabolism in *Plasmopara viticola-*infected grapevine leaves. *Mol. Plant Microbe Interact.* 24 1061–1073. 10.1094/MPMI-02-11-0040 21649510

[B27] Gessler C. Pertot I. Perazzolli M. (2011). *Plasmopara viticola*: a review of knowledge on downy mildew of grapevine and effective disease management. *Phytopathol. Mediterr.* 50 3–44. 10.14601/Phytopathol_Mediterr-9360

[B28] Gindro K. Pezet R. Viret O. (2003). Histological study of the responses of two *Vitis vinifera* cultivars (resistant and susceptible) to *Plasmopara viticola* infections. *Plant Physiol. Biochem.* 41 846–853. 10.1016/S0981-9428(03)00124-4

[B29] Halliwell B. (2006). Reactive species and antioxidants. Redox biology is a fundamental theme of aerobic life. *Plant Physiol.* 141 312–322. 10.1104/pp.106.077073 16760481 PMC1475431

[B30] Heil M. Baldwin I. T. (2002). Fitness costs of induced resistance: emerging experimental support for a slippery concept. *Trends Plant Sci.* 7 61–67. 10.1016/S1360-1385(01)02186-0 11832276

[B31] Jermini M. Blaise P. Gessler C. (2010). Influence of *Plasmopara viticola* on gas exchange parameters on field-grown *Vitis vinifera* ‘Merlot’. *Vitis* 49 87–93.

[B32] Kamoun S. Furzer O. Jones J. D. Judelson H. S. Ali G. S. Dalio R. J. (2015). The Top 10 oomycete pathogens in molecular plant pathology. *Mol. Plant Pathol.* 16 413–434. 10.1111/mpp.12190 25178392 PMC6638381

[B33] Kortekamp A. (2006). Expression analysis of defence-related genes in grapevine leaves after inoculation with a host and a non-host pathogen. *Plant Physiol. Biochem*. 44 58–67. 10.1016/j.plaphy.2006.01.008 16531058

[B34] Kortekamp A. Wind R. Zyprian E. (1997). The role of callose deposits during infection of two downy mildew-tolerant and two -susceptible *Vitis* cultivars. *Vitis* 36 103–104.

[B35] Kortekamp A. Zyprian E. (2003). Characterization of *Plasmopara*-resistance in grapevine using *in vitro* plants. *J. Plant Physiol.* 160 1393–1400. 10.1078/0176-1617-01021 14658393

[B36] Liu R. Wang L. Zhu J. Chen T. Wang Y. Xu Y. (2015). Histological responses to downy mildew in resistant and susceptible grapevines. *Protoplasma* 252 259–270. 10.1007/s00709-014-0677-1 25027553

[B37] Machado E. C. Schmidt P. T. Medina C. L. Ribeiro R. V. (2005). Respostas da fotossíntese de três espécies de citros a fatores ambientais. *Pesq. Agropec. Bras.* 40 1161–1170. 10.1590/S0100-204X2005001200002

[B38] Marguerit E. Boury C. Manicki A. Donnart M. Butterlin G. Némorin A. (2009). Genetic dissection of sex determinism, inflorescence morphology and downy mildew resistance in grapevine. *Theor. Appl. Genet.* 118 1261–1278. 10.1007/s00122-009-0979-4 19238349

[B39] Maxwell K. Johnson G. N. (2000). Chlorophyll fluorescence – A practical guide. *J. Exp. Bot.* 51 659–668. 10.1093/jexbot/51.345.659 10938857

[B40] Merdinoglu D. Wiedeman-Merdinoglu S. Coste P. Dumas V. Haetty S. Butterlin G. (2003). Genetic analysis of Downy Mildew resistance derived from *Muscadinia rotundifolia*. *Acta Hortic.* 603 451–456. 10.17660/ActaHortic.2003.603.57

[B41] Moriondo M. Orlandini S. Giuntoli A. Bindi M. (2005). The effect of downy and powdery mildew on grapevine (*Vitis vinifera* L.) leaf gas exchange. *J. Phytopathol.* 153 350–357. 10.1111/j.1439-0434.2005.00984.x

[B42] Nakano Y. Asada K. (1981). Hydrogen peroxide is scavenged by ascorbate-specific peroxidase in spinach chloroplasts. *Plant Cell Physiol.* 22 867–880. 10.1093/oxfordjournals.pcp.a076232

[B43] Nascimento R. Maia M. Ferreira A. E. Silva A. B. Freire A. P. Cordeiro C. (2019). Early stage metabolic events associated with the establishment of *Vitis vinifera* – *Plasmopara viticola* compatible interaction. *Plant Physiol. Biochem.* 137 1–13. 10.1016/j.plaphy.2019.01.026 30710794

[B44] Nascimento-Gavioli M. C. A. Agapito-Tenfen S. Z. Nodari R. O. Welter L. J. Mora F. D. S. Saifert L. (2017). Proteome of *Plasmopara viticola*-infected *Vitis vinifera* provides insights into grapevine *Rpv1/Rpv3* pyramided resistance to downy mildew. *J Proteomics* 151 264–274. 10.1016/j.jprot.2016.05.024 27235723

[B45] Nogueira Júnior A. F. Ribeiro R. V. Appezzato-da-Glória B. Soares M. K. Rasera J. B. Amorim L. (2017). *Phakopsora euvitis* causes unusual damage to leaves and modifies carbohydrate metabolism in grapevine. *Front. Plant Sci.* 8:1675. 10.3389/fpls.2017.01675 29018470 PMC5623187

[B46] Nogueira Júnior A. F. Ribeiro R. V. Marcos F. C. C. Amorim L. (2019). Virtual lesions and photosynthetic damage caused by *Plasmopara viticola* in *Vitis labrusca*. *Eur. J. Plant Pathol.* 155 545–555. 10.1007/s10658-019-01791-2

[B47] Parlevliet J. E. (1979). Components of resistance that reduce the rate of epidemic development. *Ann. Rev. Phytopathol.* 17 203–222. 10.1146/annurev.py.17.090179.001223

[B48] Peressotti E. Wiedemann-Merdinoglu S. Delmotte F. Bellin D. Di Gaspero G. Testolin R. (2010). Breakdown of resistance to grapevine downy mildew upon limited deployment of a resistant variety. *BMC Plant Biol.* 10:147. 10.1186/1471-2229-10-147 20633270 PMC3095292

[B49] Purrington C. B. (2000). Costs of resistance. *Curr. Opin. Plant Biol.* 3 305–308. 10.1016/S1369-5266(00)00085-6 10873846

[B50] Richter H. Pezet R. Viret O. Gindro K. (2006). Characterization of 3 new partial stilbene synthase genes out of over 20 expressed in *Vitis vinifera* during the interaction with *Plasmopara viticola*. *Physiol. Mol. Plant Pathol.* 67 248–260. 10.1016/j.pmpp.2006.03.001

[B51] Rossi V. Caffi T. Gobbin D. (2013). Contribution of molecular studies to botanical epidemiology and disease modelling: grapevine downy mildew as a case-study. *Eur. J. Plant Pathol.* 135 641–654. 10.1007/s10658-012-0114-2

[B52] Scharte J. Schön H. Weis E. (2005). Photosynthesis and carbohydrate metabolism in tobacco leaves during an incompatible interaction with *Phytophthora nicotianae*. *Plant Cell Environ.* 28 1421–1435. 10.1111/j.1365-3040.2005.01380.x

[B53] Schwander F. Eibach R. Fechter I. Hausmann L. Zyprian E. Töpfer R. (2012). Rpv10: a new locus from the Asian *Vitis* gene pool for pyramiding downy mildew resistance loci in grapevine. *Theor. Appl. Genet.* 124 163–176. 10.1007/s00122-011-1695-4 21935694

[B54] Slaughter A. R. Hamiduzzaman M. M. Gindro K. Neuhaus J. M. Mauch-Mani B. (2008). Beta-aminobutyric acid-induced resistance in grapevine against downy mildew: involvement of pterostilbene. *Eur. J. Plant Pathol.* 122 185–195. 10.1007/s10658-008-9285-2

[B55] Smedegaard-Petersen V. Tolstrup K. (1985). The limiting effect of disease resistance on yield. *Ann. Rev. Phytopathol.* 23 475–490. 10.1146/annurev.py.23.090185.002355

[B56] Swarbrick P. J. Schulze-Lefert P. Scholes J. D. (2006). Metabolic consequences of susceptibility and resistance (race-specific and broad-spectrum) in barley leaves challenged with powdery mildew. *Plant Cell Environ.* 29 1061–1076. 10.1111/j.1365-3040.2005.01472.x 17080933

[B57] Thordal-Christensen H. Zhang Z. Wei Y. Collinge D. B. (1997). Subcellular localization of H_2_O_2_ in plants. *H*_2_O_2_ accumulation in papillae and hypersensitive response during the barley-powdery mildew interaction. *Plant J.* 11 1187–1194. 10.1046/j.1365-313X.1997.11061187.x

[B58] Trouvelot S. Varnier A.-L. Allègre M. Mercier L. Baillieul F. Arnould C. (2008). A β-1,3 glucan sulfate induces resistance in grapevine against *Plasmopara viticola* through priming of defense responses, including HR-like cell death. *Mol. Plant-Microbe Interact.* 21 232–243. 10.1094/MPMI-21-2-0232 18184067

[B59] Vale F. X. R. Fernandes Filho E. I. F. Liberato J. R. (2001). “QUANT – a software for plant disease severity assessment,” in *Proceedings of the 8th International Congress of Plant Pathology*. Christchurch, NZ, 105. 10.1094/mpmi-21-2-0232

[B60] Venuti S. Copetti D. Foria S. Falginella L. Hoffmann S. Bellin D. (2013). Historical introgression of the downy mildew resistance gene Rpv12 from the Asian species *Vitis amurensis* into grapevine varieties. *PLoS ONE* 8:e61228. 10.1371/journal.pone.0061228 23593440 PMC3625174

[B61] Vezzulli S. Malacarne G. Masuero D. Vecchione A. Dolzani C. Goremykin V. (2019). The *Rpv*3-3 haplotype and stilbenoid induction mediate downy mildew resistance in a grapevine interspecific population. *Front. Plant Sci.* 10:234. 10.3389/fpls.2019.00234 30894868 PMC6414455

[B62] Walters D. R. Boyle C. (2005). Induced resistance and allocation cost: what is the impact of pathogen challenge? *Physiol. Mol. Plant Pathol.* 66 40–44. 10.1016/j.pmpp.2005.04.002

[B63] Welter L. Göktürk-Baydar N. Akkurt M. Maul E. Eibach R. Töpfer R. (2007). Genetic mapping and localization of quantitative trait loci affecting fungal disease resistance and leaf morphology in grapevine (*Vitis vinifera* L). *Mol. Breed.* 20 359–374. 10.1007/s11032-007-9097-7

[B64] Wilcox W. F. Gubler W. D. Uyemoto J. K. (2015). *Compendium of Grape Diseases, Disorders and Pests.* Saint Paul, MN: APS Press.

[B65] Wolff S. P. (1994). Ferrous ion oxidation in presence of ferric ion indicator xylenol orange for measurement of hydroperoxides. *Methods Enzymol.* 233 182–189. 10.1016/S0076-6879(94)33021-2

