# Supplementary material for: Photosynthetic Cost Associated With Induced Defense to Plasmopara viticola in Grapevine
Source: Front Plant Sci. 2020 Mar 19;11:235. doi: 10.3389/fpls.2020.00235 (PMC7098430; doi:10.3389/fpls.2020.00235)
Supplement: Supplementary file 1 [file Data_Sheet_1.docx]

Supplementary Material

*Photosynthetic cost associated with induced defense to Plasmopara viticola in grapevine*

Antonio F. Nogueira Júnior, Merle Tränkner, Rafael V. Ribeiro, Andreas von Tiedemann, Lilian Amorim,*

* Correspondence: Corresponding Author: lilian.amorim@usp.br

## Supplementary Figures


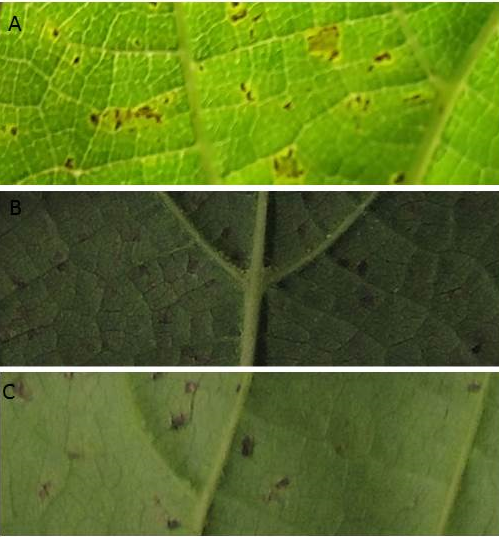


**Supplementary Figure 1.** Cell death-like hypersensitive reactions 4 days after inoculation with *Plasmopara viticola* in the resistant cultivars Solaris (A), Cabernet Blanc (B) and Phoenix (C).


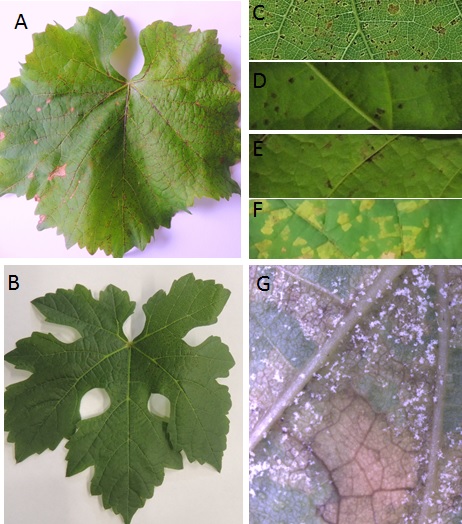


**Supplementary Figure 2.** Cell death-like hypersensitive reactions 12 days after inoculation with *Plasmopara viticola* in the resistant cultivars Solaris (A, C), Cabernet Blanc (B, D) and Phoenix (E), and downy mildew symptoms (F) and abundant sporulation of *P. viticola* (G) in the susceptible cultivar Riesling. Images from C to F represent examples of leaf areas where the gas exchange measurements were performed.


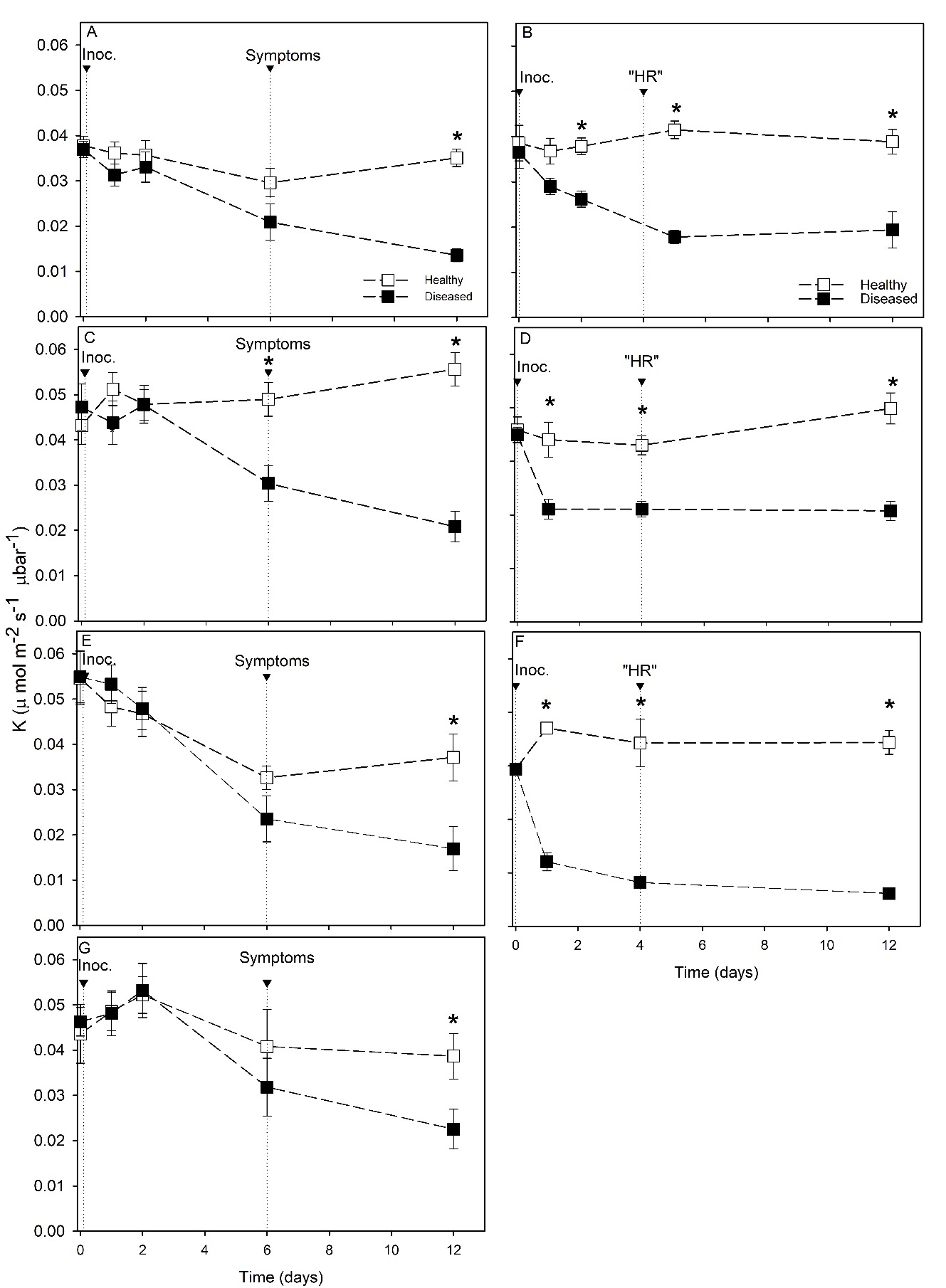


**Supplementary Figure 3.** Instantaneous carboxylation efficiency (*k*) in *Vitis* spp*.* cultivars susceptible (A, C, E, G) and resistant (B, D, F) to downy mildew before and after inoculation with *Plasmopara viticola*. Inoculation was performed after the first gas exchange measurement. Cell death-like hypersensitive reactions “HR” were observed in Solaris (B), Cabernet Blanc (D) and Phoenix (F) four days after inoculation. Downy mildew symptoms were observed in Riesling (A), Niagara Rosada (C), Merlot (E) and Moscato (G) six days after inoculation. The average values of healthy and diseased leaves were compared using the Student’s *t* test for each cultivar (n=6, ± SE), and * indicates significant differences (*p*<0.05).


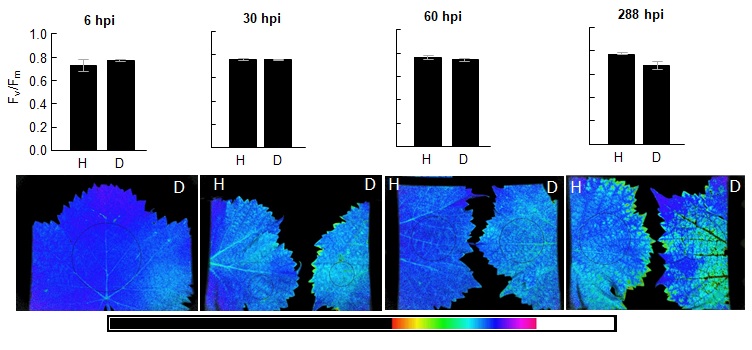


**Supplementary Figure 4.** The maximum PSII quantum efficiency (F_v_/F_m_) and chlorophyll fluorescence images showing F_v_/F_m_ in grapevine leaves of cv. Solaris after 6, 30, 60, and 288 hours post infection (hpi) with *Plasmopara viticola* (D) and healthy (H) leaves. Circles indicate the area used for calculation of F_v_/F_m_. Leaves were dark-adapted prior to measurements. The false color code depicted at the bottom of images represents the range of 0 (black) to 1 (white).

**
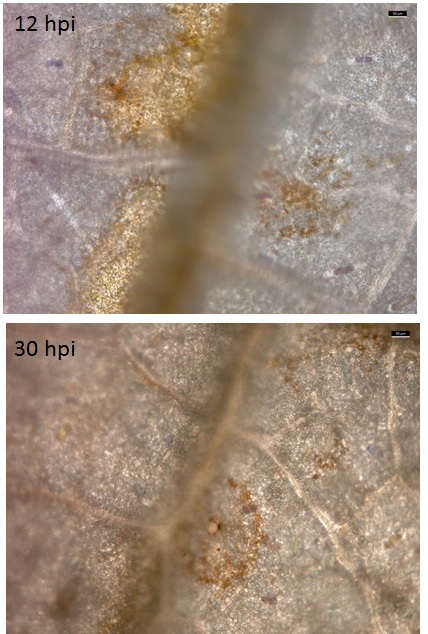
**

**Supplementary Figure 5.** H_2_O_2_ detection by DAB staining at 12 and 30 hai with *Plasmopara viticola* in cv. Solaris. Scale bars = 50 µm**.**


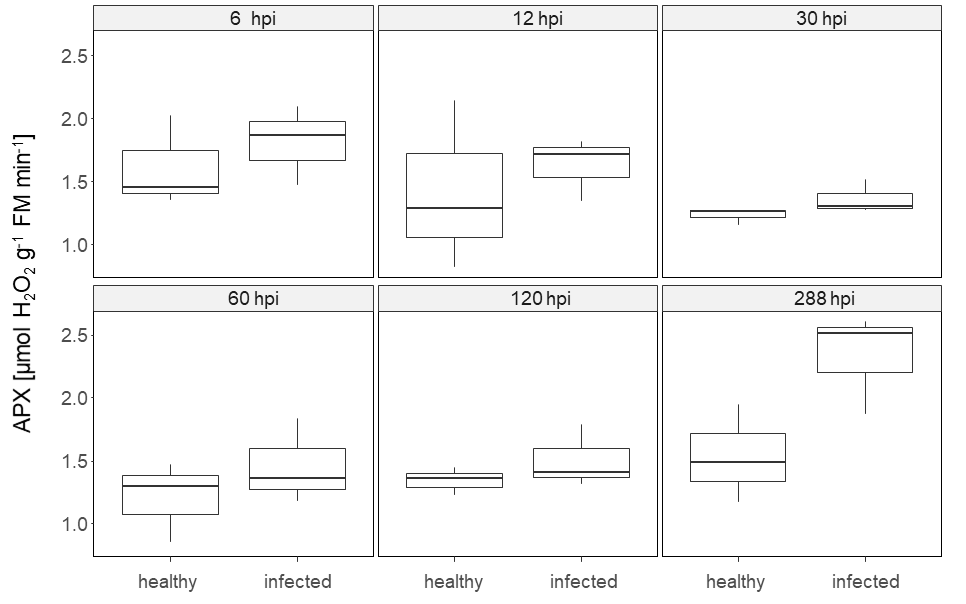


**Supplementary Figure 6.** Time-course of ascorbate peroxidase (APX) activity in healthy and *Plasmopara viticola* infected leaves after inoculation in cv. Solaris.

**Supplementary Table 1**. *P* values of Shapiro – Wilk´s normality test for the data of net photosynthetic rate (*A*), stomatal conductance (*g*_s_), intercellular CO_2_ concentration (*C*_i_) and transpiration rate (E). *P* values > 0.05 are in blue and represent data with normal distribution. *P* values in red represent data with no normal distribution and the transformations utilised are below the value.

| Cultivars | *Shapiro - Wilk`s p* value for normality | | | |
| --- | --- | --- | --- | --- |
|  | ***A*** | ***g*_s_** | ***C*_i_** | ***E*** |
| Solaris | 0.61 | 0.43 | 0.01 | 0.07 |
|  |  |  | √(√(x +1))^a^ |  |
| Cabernet | 0.24 | 0.71 | 0.08 | 0.63 |
|  |  |  |  |  |
| Phoenix | 0.06 | 0.14 | 0.61 | 0.03 |
|  |  |  |  | √(x +1) |
| Riesling | 0.06 | 0.005 | 0.09 | 0.02 |
|  |  | √(x +1) |  | (√(x))*5 |
| Niagara | 0.32 | 0.06 | 0.43 | 0.02 |
|  |  |  |  | (√(x))*5 |
| Merlot | 0.06 | 0.18 | 0.34 | 0.03 |
|  |  |  |  | (√(x))*5 |
| Moscato | 0.74 | 0.002 | 0.79 | 0.02 |
|  |  | √(x +1) |  | (√(x))*5 |

^a^ √ means square root.
